# Cotrimoxazole prophylaxis decreases tuberculosis risk among Asian patients with HIV

**DOI:** 10.1002/jia2.25264

**Published:** 2019-03-29

**Authors:** Stephane Wen‐Wei Ku, Awachana Jiamsakul, Kedar Joshi, Mark Kristoffer Ungos Pasayan, Alvina Widhani, Romanee Chaiwarith, Sasisopin Kiertiburanakul, Anchalee Avihingsanon, Penh Sun Ly, Nagalingeswaran Kumarasamy, Cuong D Do, Tuti P Merati, Kinh Van Nguyen, Adeeba Kamarulzaman, Fujie Zhang, Man Po Lee, Jun Yong Choi, Junko Tanuma, Suwimon Khusuwan, Benedict Lim Heng Sim, Oon Tek Ng, Winai Ratanasuwan, Jeremy Ross, Wing‐Wai Wong, TREAT Asia HIV Observational Database (TAHOD) of IeDEA Asia‐Pacific, PS Ly, V Khol, FJ Zhang, HX Zhao, N Han, MP Lee, PCK Li, W Lam, YT Chan, N Kumarasamy, S Saghayam, C Ezhilarasi, S Pujari, K Joshi, S Gaikwad, A Chitalikar, S Sangle, V Mave, I Marbaniang, DN Wirawan, F Yuliana, E Yunihastuti, D Imran, A Widhani, J Tanuma, S Oka, T Nishijima, JY Choi, Na S, JM Kim, BLH Sim, YM Gani, NB Rudi, A Kamarulzaman, SF Syed Omar, S Ponnampalavanar, I Azwa, R Ditangco, MK Pasayan, ML Mationg, WW Wong, SWW Ku, PC Wu, OT Ng, PL Lim, LS Lee, Z Ferdous, A vihingsanon, S Gatechompol, P Phanuphak, C Phadungphon, S Kiertiburanakul, A Phuphuakrat, L Chumla, N Sanmeema, R Chaiwarith, T Sirisanthana, W Kotarathititum, J Praparattanapan, S Khusuwan, P Kantipong, P Kambua, W Ratanasuwan, R Sriondee, KV Nguyen, HV Bui, DTH Nguyen, DT Nguyen, CD Do, AV Ngo, LT Nguyen, AH Sohn, JL Ross, B Petersen, DA Cooper, MG Law, A Jiamsakul, D Rupasinghe

**Affiliations:** ^1^ Division of Infectious Diseases Department of Medicine Taipei Veterans General Hospital Taipei Taiwan; ^2^ Division of Infectious Diseases Department of Medicine Taipei City Hospital Renai Branch Taipei Taiwan; ^3^ The Kirby Institute UNSW Sydney NSW Australia; ^4^ Institute of Infectious Diseases Pune India; ^5^ Research Institute for Tropical Medicine Manila Philippines; ^6^ Working Group on AIDS Faculty of Medicine University of Indonesia/Cipto Mangunkusumo Hospital Jakarta Indonesia; ^7^ Research Institute for Health Sciences Chiang Mai University Chiang Mai Thailand; ^8^ Faculty of Medicine Ramathibodi Hospital Mahidol University Bangkok Thailand; ^9^ Faculty of Medicine Chulalongkorn University and HIV‐NAT/Thai Red Cross AIDS Research Centre Bangkok Thailand; ^10^ National Center for HIV/AIDS Dermatology & STDs, and University of Health Sciences Phnom Penh Cambodia; ^11^ Chennai Antiviral Research and Treatment Clinical Research Site (CART CRS) YRGCARE Medical Centre VHS Chennai India; ^12^ Bach Mai Hospital Hanoi Vietnam; ^13^ Faculty of Medicine Udayana University & Sanglah Hospital Bali Indonesia; ^14^ National Hospital for Tropical Diseases Hanoi Vietnam; ^15^ University Malaya Medical Centre Kuala Lumpur Malaysia; ^16^ Beijing Ditan Hospital Capital Medical University Beijing China; ^17^ Queen Elizabeth Hospital Hong Kong SAR China; ^18^ Department of Internal Medicine Yonsei University College of Medicine Seoul South Korea; ^19^ AIDS Research Institute Yonsei University College of Medicine Seoul South Korea; ^20^ National Center for Global Health and Medicine Tokyo Japan; ^21^ Chiangrai Prachanukroh Hospital Chiang Rai Thailand; ^22^ Hospital Sungai Buloh Sungai Buloh Malaysia; ^23^ Tan Tock Seng Hospital Tan Tock Seng Singapore; ^24^ Faculty of Medicine Siriraj Hospital Mahidol University Bangkok Thailand; ^25^ TREAT Asia amfAR – The Foundation for AIDS Research Bangkok Thailand

**Keywords:** cotrimoxazole, sulphamethoxazole/trimethoprim, tuberculosis, HIV, AIDS, Asia, cohort studies, prophylaxis

## Abstract

**Introduction:**

Cotrimoxazole (CTX) is recommended as prophylaxis against *Pneumocystis jiroveci* pneumonia, malaria and other serious bacterial infections in HIV‐infected patients. Despite its *in vitro* activity against *Mycobacterium tuberculosis*, the effects of CTX preventive therapy on tuberculosis (TB) remain unclear.

**Methods:**

Adults living with HIV enrolled in a regional observational cohort in Asia who had initiated combination antiretroviral therapy (cART) were included in the analysis. Factors associated with new TB diagnoses after cohort entry and survival after cART initiation were analysed using Cox regression, stratified by site.

**Results:**

A total of 7355 patients from 12 countries enrolled into the cohort between 2003 and 2016 were included in the study. There were 368 reported cases of TB after cohort entry with an incidence rate of 0.99 per 100 person‐years (/100 pys). Multivariate analyses adjusted for viral load (VL), CD4 count, body mass index (BMI) and cART duration showed that CTX reduced the hazard for new TB infection by 28% (HR 0.72, 95% CI l 0.56, 0.93). Mortality after cART initiation was 0.85/100 pys, with a median follow‐up time of 4.63 years. Predictors of survival included age, female sex, hepatitis C co‐infection, TB diagnosis, HIV VL, CD4 count and BMI.

**Conclusions:**

CTX was associated with a reduction in the hazard for new TB infection but did not impact survival in our Asian cohort. The potential preventive effect of CTX against TB during periods of severe immunosuppression should be further explored.

## 1 Introduction

Cotrimoxazole (CTX) has been recommended as prophylaxis against *Pneumocystis jiroveci* pneumonia (PJP), toxoplasmosis, malaria and other serious bacterial infections in adults with severe or advanced HIV clinical disease, with a CD4 count less than 350 cells/μL or less than 200 cells/μL (depending on region), by the World Health Organization (WHO) 1, 2. Although antituberculous effects of sulphonamides were identified in the late 1930s, their use in treatment against tuberculosis (TB) was basically forgotten because of the toxicity of the early sulphonamides and that isoniazid and streptomycin were stronger drugs 3, 4. However, in a previous study, the majority of clinical isolates of *Mycobacterium tuberculosis* (Mtb) from different patients were found sensitive to CTX 5. Further studies demonstrated sulphamethoxazole, instead of trimethoprim, had *in vitro* bacteriostatic activity against Mtb 6, 7. Not until recently had their potential role in treatment or prophylaxis against TB been a major consideration in their use in patients with HIV who may be taking CTX for PJP prophylaxis. The first randomized controlled trial conducted in Côte d'Ivoire has demonstrated that daily CTX prophylaxis was well tolerated and significantly decreased mortality and hospital admission rates in HIV‐infected patients with pulmonary tuberculosis 8. A recent randomized controlled trial in Cambodia has found that absence of CTX prophylaxis in HIV‐infected adult patients with smear‐positive tuberculosis was associated with an increased rate of late mortality 9. Several cohort studies also found a decreased risk of death in TB/HIV‐coinfected patients receiving CTX preventive therapy in resource‐limited settings 10, 11. A Swiss HIV Cohort Study suggested CTX reduced the incidence of TB among HIV‐infected persons, and although a recent case–control study in Ethiopia also found CTX had a protective effect against TB 12, 13, findings from a South African cohort study did not support a preventive effect 14. This study aims to examine the incidence of TB and survival in HIV‐infected patients receiving and not receiving CTX prophylaxis in a regional observational cohort in Asia.

## 2 Methods

### 2.1 Study population

Patients were included if they were enrolled in the adult (age ≥ 18 years) TREAT Asia HIV Observational Database (TAHOD) of IeDEA Asia‐Pacific from 2003 and had initiated combination antiretroviral therapy (cART). Patients who had not initiated cART or those who initiated with mono/dual therapy were excluded. All patients were analysed based on the intention‐to‐treat approach where patients were considered to be on cART for the entire follow‐up time after cART had been initiated, regardless of whether treatment interruptions had occurred.

#### 2.1.1 Analysis (i): Factors associated with first TB diagnosis after TAHOD entry

Patients were included if they had at least one day of follow‐up after cohort entry. TB diagnosis is defined as definitive when there is isolation (or culture) of Mtb complex* *from a clinical specimen. TB is presumptively diagnosed when there is demonstration of acid‐fast bacilli in a clinical specimen, or in a histopathological lesion when a culture is not available, in a person with signs or symptoms compatible with tuberculosis; or evidence of resolution of disease where treatment with two or more antituberculosis medications have been prescribed and follow‐up has been instigated. These diagnostic criteria have been used in TAHOD and published previously elsewhere 15, 16, 17. A TB diagnosis up to seven days after cohort entry was included as prior TB events 16. Patients without evidence of TB diagnosis during follow‐up but have died with TB as the reported cause of death were also included as being diagnosed with TB on the date of death. Risk time for TB started from the date of cohort entry and ended on the date of TB diagnosis, defined as the outcome of this analysis. Factors associated with TB diagnosis after cohort entry was analysed using Cox regression, stratified by site. Patients who did not develop TB were censored at date of last follow‐up. A TB diagnosis event could occur at any time either before or after cART initiation, but post‐cohort enrolment. Time‐fixed covariates included in the regression analysis were age at cohort entry, sex, HIV exposure category, hepatitis B/C co‐infection and a history of prior TB events. Time‐updated covariates were viral load (VL), CD4 count, body mass index (BMI), cART duration, and CTX and isoniazid use. Age, VL, CD4 and BMI were included as categorical variables based on clinically relevant categories for our patient group as well as taking into consideration the distribution of our data. Age was categorized into 10‐year groups to illustrate the effects of hazard ratios (HRs) for each decade between 30 and 50 years of age. Viral load was categorized to represent different levels of detectable and undetectable viral loads 18, 19 **.** CD4 cell count represented the different low levels below 200 cells/μL at which CTX was initiated. BMI categories were grouped as “overweight” and “not overweight.” If CTX or isoniazid was initiated in the 60 days prior to the diagnosis of a new TB diagnosis, the TB episode was coded as not exposed to CTX or isoniazid because these drugs may have been started as a result of TB symptoms rather than as preventative measures 14.

#### 2.1.2 Analysis (ii): Survival time after cART initiation

Patients who had at least one day of follow‐up after cohort entry or cART initiation (whichever occurred last) were eligible for inclusion in the analysis. Risk time for mortality after cART initiation began from the date of cART initiation and ended on the date of death or date of last follow‐up. For patients who initiated cART prior to cohort entry, survival time was left‐truncated at cohort entry. Survival time was analysed using Cox regression, stratified by site. Time‐fixed covariates were age at cART initiation, sex, HIV exposure category and hepatitis B/C co‐infection. Time‐updated covariates were new TB diagnosis after cohort entry, VL, CD4 count, BMI, and CTX and isoniazid use, which were coded in the same way as Analysis (i).

All regression models were fitted using a backward stepwise selection process. Covariates significant at *p *<* *0.10 in the univariate analyses were chosen for inclusion in the multivariate models. Covariates with *p *<* *0.05 were considered statistically significant in the final model. Non‐significant covariates were presented in the tables adjusted for significant predictors; however, they did not form part of the final multivariate model. Crude incidence rates for TB diagnosis and mortality were plotted for CTX by time‐updated CD4 cell count category. Cox proportional hazards (PH) assumption was tested using Schoenfeld residuals and log–log plots.

### 2.2 Sensitivity analyses

Several sensitivity analyses were performed to further assess the association with TB diagnosis:

Sensitivity analysis (a) and (b): TB diagnosis was further classified as presumptive or definitive. Those with unreported TB classification were grouped into “presumptive” TB cases. Fine and Gray competing risk regression, adjusted for site, was used to analyse factors associated with presumptive TB (sensitivity analysis (a)) and definitive TB (sensitivity analysis (b)).

Sensitivity analysis (c) and (d): Risk factors for TB diagnosis was analysed separately for males (sensitivity analysis (c) and females (sensitivity analysis (d)), using Cox regression methods, stratified by site.

Ethics approvals were obtained from the local institutional review boards of each TAHOD‐participating site, the data management and biostatistics centre (UNSW Sydney Ethics Committee) and the coordinating centre (TREAT Asia/amfAR). The informed consent was obtained or waived according to the regulation from the local institutional review boards of each TAHOD‐participating site. All data management and statistical analyses were performed using SAS software version 9.4 (SAS Institute Inc., Cary, NC, USA) and Stata software version 14.1 (StataCorp, College Station, TX, USA).

## 3 Results

**Figure 1:**
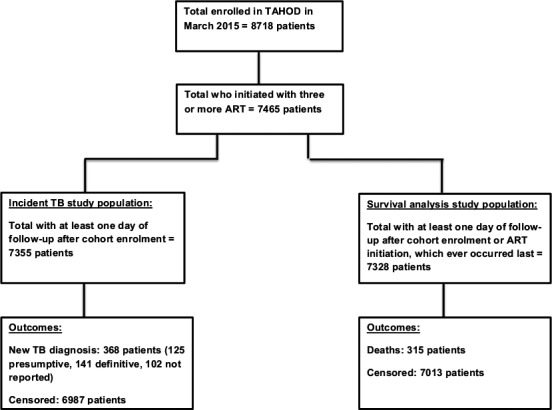
Flow diagram

A total of 8718 patients were enrolled in TAHOD as of March 2015. There were 7465 patients (86%) who had initiated with three or more cART. Of the 7465 patients, 7355 had at least one day of follow‐up from cohort enrolment and were included in Analysis (i). A total of 7328 patients were included in Analysis (ii) as they had at least one day of follow‐up from the latter of cohort enrolment date or date of cART initiation (Figure 1).

### 3.1 Analysis (i): Factors associated with first TB diagnosis after cohort entry

A total of 7355 patients from Cambodia, China, Hong Kong SAR, India, Indonesia, Japan, Malaysia, Philippines, Singapore, South Korea, Taiwan, Thailand and Vietnam were included between 2003 and 2015. Of the 7355 patients, 5150 (70%) were male, 3205 (44%) were aged between 31 and 40 years, 4682 (64%) acquired HIV through heterosexual exposure, 5050 (89% of 5644 tested) had no hepatitis B co‐infection, 4544 (85% of 5331 tested) had no hepatitis C co‐infection and 5856 (80%) had no prior TB diagnosis (Table 1). There were 368 (5%) new cases of TB reported after cohort entry with an incidence rate of 0.99/100 person‐years (100 pys). The median follow‐up time up to the TB diagnosis event was 4.6 years (interquartile range (IQR) 2.8 to 7.0). Of the 368 TB cases, 125 (34%) were presumptive, 141 (38%) were definitive and 102 (28%) did not report TB diagnosis category. There was a total of 26 patients who had died due to TB, but without prior evidence of TB diagnosis recorded in the database after cohort enrolment. The median CD4 cell count at time of TB diagnosis was 164 cells/μL (IQR 58 to 287) for presumptive TB, 205 cells/μL (IQR 89 to 336) for definitive TB and 162 cells/μL (IQR 25 to 293) for unknown category TB cases.

**Table 1:** Factors associated with TB diagnosis after cohort entry

	No. of patients^b^	No. of TB diagnosis	Rate (/100 pys)	Univariate		Multivariate^a^	
				HR (95% CI)	*p*‐value^c^	HR (95% CI)	*p*‐value^c^^,^ d
Total	7355	368	0.99				
Age at TAHOD entry (years)							
≤30	1982	100	1.07	1	0.944	1	0.118
31 to 40	3205	175	1.05	1.13 (0.88, 1.45)	0.328	1.30 (1.01, 1.67)	0.042
41 to 50	1513	73	0.94	1.14 (0.83, 1.55)	0.418	1.47 (1.07, 2.01)	0.018
>50	655	20	0.59	0.82 (0.50, 1.33)	0.422	1.05 (0.64, 1.73)	0.842
Sex							
Male	5150	280	1.08	1		1	
Female	2205	88	0.78	0.76 (0.59, 0.98)	0.031	0.79 (0.62, 1.01)	0.064
HIV exposure							
Heterosexual contact	4682	253	1.03	1	0.125	1	0.501
Homosexual contact	1580	50	0.63	0.77 (0.52, 1.12)	0.176	0.88 (0.60, 1.30)	0.527
Injecting drug use	555	41	1.99	1.49 (0.97, 2.29)	0.067	1.34 (0.87, 2.06)	0.182
Other/Unknown	538	24	0.88	0.90 (0.57, 1.42)	0.661	0.96 (0.61, 1.51)	0.870
Hepatitis B co‐infection							
Negative	5050	234	0.9	1		1	
Positive	594	26	0.86	1.11 (0.74, 1.67)	0.624	1.07 (0.71, 1.62)	0.738
Not tested	1711	108	1.33				
Hepatitis C co‐infection							
Negative	4544	186	0.77	1		1	
Positive	787	42	1.27	1.38 (0.94, 2.04)	0.102	1.28 (0.86, 1.90)	0.217
Not tested	2024	140	1.45				
Prior TB							
No	5856	239	0.8	1		1	
Yes	1499	129	1.77	1.34 (1.06, 1.68)	0.013	1.22 (0.97, 1.54)	0.094
Viral load (copies/mL)							
<400	–	76	0.3	1	<0.001	1	**<0.001**
400 to 999	–	3	0.55	1.45 (0.45, 4.63)	0.534	1.02 (0.32, 3.26)	0.979
1000 to 4999	–	9	1.24	3.07 (1.51, 6.26)	0.002	2.18 (1.06, 4.48)	**0.034**
≥5000	–	100	2.93	5.35 (3.77, 7.60)	<0.001	2.38 (1.63, 3.47)	**<0.001**
Missing	–	180	2.47				
CD4 (cells/μL)							
≤50	–	78	9.91	1	<0.001	1	**<0.001**
51 to 100	–	35	4.05	0.49 (0.33, 0.74)	0.001	0.57 (0.38, 0.86)	**0.007**
101 to 200	–	77	2.18	0.33 (0.24, 0.46)	<0.001	0.42 (0.30, 0.58)	**<0.001**
>200	–	158	0.5	0.09 (0.06, 0.12)	<0.001	0.11 (0.08, 0.15)	**<0.001**
Missing	–	20	3.58				
BMI (kg/m^2^)							
<25	–	265	1.09	1		1	
≥25	–	27	0.46	0.39 (0.26, 0.58)	<0.001	0.46 (0.31, 0.69)	**<0.001**
Missing	–	76	1.09				
cART duration							
Prior to cART initiation	–	53	3.2	1	<0.001	1	**<0.001**
<6 months	–	84	5.43	1.72 (1.15, 2.58)	0.008	1.08 (0.70, 1.66)	0.727
6 to 12 months	–	29	1.45	0.54 (0.32, 0.89)	0.015	0.47 (0.28, 0.80)	**0.005**
>12 months	–	202	0.63	0.33 (0.24, 0.46)	<0.001	0.40 (0.28, 0.57)	**<0.001**
Cotrimoxazole use							
No	–	231	0.75	1		1	
Yes	–	137	2.13	1.71 (1.36, 2.15)	<0.001	0.72 (0.56, 0.93)	**0.011**
Isoniazid use							
No	–	353	0.98	1		1	
Yes	–	15	1.31	1.20 (0.69, 2.08)	0.520	1.12 (0.64, 1.97)	0.684

^a^Non‐significant covariates were presented in the final model adjusted for the significant covariates; however, they did not form part of the final model; ^b^viral load, CD4, BMI, cART duration, cotrimoxazole and isoniazid use are time‐updated variables; ^c^global *p*‐values for age, VL and CD4 are tests for trend. All other global *p*‐values are tests for heterogeneity excluding missing values; ^d^ *p*‐values in bold represent significant covariates in the final model.

In the univariate analyses, sex (*p *=* *0.031), VL (*p *<* *0.001), CD4 count (*p *<* *0.001), BMI (*p *<* *0.001), cART duration (*p *<* *0.001) and CTX use (*p *<* *0.001) were significantly associated with incident TB diagnosis. In multivariate analyses, higher VL (1000 to 4999 copies/mL HR 2.18, 95% CI (1.06 to 4.48), *p *=* *0.034; and ≥ 5000 copies/mL HR 2.38, 95% CI (1.63 to 3.47), *p *<* *0.001) were associated with increased hazard of developing TB compared to VL < 400 copies/mL. Conversely, higher CD4 count (51 to 100 cells/μL HR 0.57, 95% CI (0.38 to 0.86), *p *=* *0.007; 101 to 200 cells/μL HR 0.42, 95% CI (0.30 to 0.58), *p *<* *0.001; and > 200 cells/μL HR 0.11, 95% CI (0.08 to 0.15), *p *<* *0.001) was associated with reduced hazard for TB compared to CD4 ≤ 50 cells/μL. Other factors associated with reduction in hazard for development of TB were BMI ≥ 25 kg/m^2^ (HR 0.46, 95% CI (0.31 to 0.69), *p *<* *0.001) compared to BMI < 25 kg/m^2^; longer cART durations (6 to 12 months HR 0.47, 95% CI (0.28 to 0.80), *p *=* *0.005; and > 12 months HR 0.40, 95% CI (0.28 to 0.57), *p *<* *0.001) compared to periods prior to cART initiation; and CTX use (HR 0.72, 95% CI (0.56 to 0.93), *p *=* *0.011) (Table 1). We found that the hazard for CTX was reversed after adjusting for CD4 count (from HR 1.71 to HR 0.72), indicating possible confounding by CD4 levels. When tested for PH assumption, it was noted that CD4 levels violated the assumption at early time periods. We have therefore stratified the analysis by both CD4 and site to account for this violation (Table S1). The multivariate results were similar to those obtained in Table 1 suggesting that the violation of the PH assumption was minor and did not have a great impact on our results.

**Figure 2:**
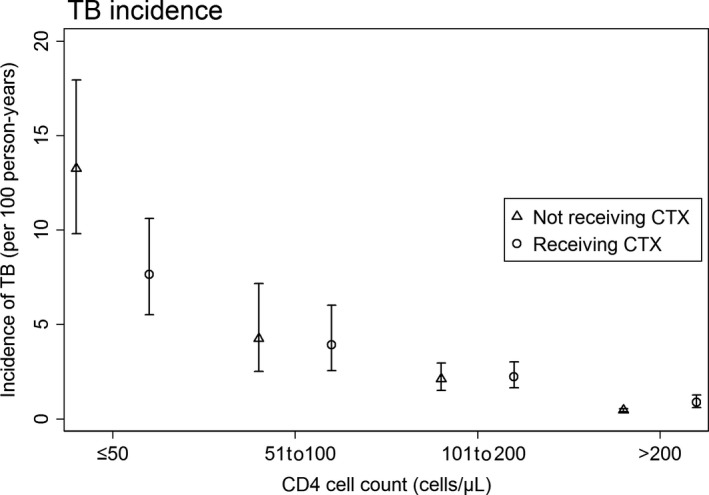
TB incidence

The crude TB incidence rates in those receiving and not receiving CTX, stratified by CD4 count, are shown in Figure 2. Overall, the incidence of TB decreased with increasing CD4 cell count. Among patients with CD4 ≤ 50 cells/μL, the incidence was lower in those receiving CTX (7.7/100 pys) compared those not receiving CTX (13.3/100 pys) (*p *=* *0.002). To explore this further, we performed a univariate analysis by including CTX and limiting the analyses to each CD4 cell count category while taking into account heterogeneity across sites. We found that CTX use at CD4 ≤ 50 cells/μL was associated with reduced hazard for TB compared to those not receiving CTX within the same CD4 ≤ 50 cells/μL category: HR = 0.37, 95% CI (0.21 to 0.65), *p *<* *0.001. No significant difference was found in those receiving CTX at CD4 51 to 100 cells/μL (HR = 0.47, 95% CI (0.19 to 1.16), *p *=* *0.102). For those taking CTX at CD4 101 to 200 cells/μL, there was a 44% reduction in the hazard for development of TB (HR = 0.56, 95% CI (0.33 to 0.94), *p *=* *0.03) compared to those no receiving CTX. No significance difference was found for among those with CD4 > 200 cells/μL HR = 1.30, 95% CI (0.85 to 2.00), *p *=* *0.222. In summary, CTX was associated with reduced hazards for TB among those with current CD4 ≤ 50 cells/μL and 101 to 200 cells/μL.

### 3.2 Analysis (ii): Survival time after cART initiation

**Figure 3:**
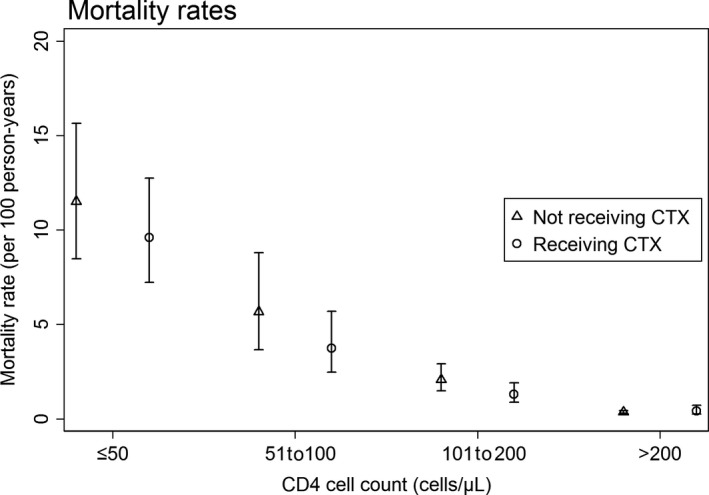
Mortality rates

A total of 7328 patients were included in the survival analysis (Table 2). The mortality rate was 0.85/100 pys, with a median follow‐up time of 4.63 years (IQR 2.80 to 6.92 years). In the adjusted model, factors associated with poorer survival were older age (41 to 50 years HR 1.49, 95% CI (1.05 to 2.13), *p *=* *0.027; and > 50 years HR 3.90, 95% CI (2.70 to 5.62), *p *<* *0.001) compared to age ≤ 30 years; being hepatitis C antibody positive (HR 1.90, 95% CI (1.33 to 2.72), *p *<* *0.001); having an incident TB diagnosis (HR 2.50, 95% CI (1.73 to 3.63), *p *<* *0.001); and having VL ≥ 5000 copies/mL (HR 1.59, 95% CI (1.09 to 2.34), *p *=* *0.017) compared to VL < 400 copies/mL. Factors associated with improved survival were female sex (HR 0.70, 95% CI (0.53 to 0.94), *p *=* *0.017); higher CD4 count (51 to 100 cells/μL HR 0.42, 95% CI (0.29 to 0.62); 101 to 200 cells/μL HR 0.19, 95% CI (0.13 to 0.28); and > 200 cells/μL HR 0.06, 95% CI (0.04 to 0.09), all *p *<* *0.001) compared to CD4 ≤ 50 cells/μL; and BMI ≥ 25 kg/m^2^ (HR 0.40, 95% CI (0.24 to 0.68), *p *=* *0.001) compared BMI < 25 kg/m^2^. Those using CTX also had improved survival; however, this effect was not statistically significant (HR 0.78, 95% CI (0.58 to 1.03, *p *=* *0.081). Crude mortality rates in those receiving and not receiving CTX, stratified by CD4 count, are shown in Figure 3. The hazard for mortality was only significantly lower in those receiving CTX at CD4 101 to 200 cells/μL (HR = 0.51, 95% CI (0.28 to 0.94), *p *=* *0.031).

**Table 2:** Survival time after cART initiation

	Number of patients^b^	Deaths	Rate (/100 pys)	Univariate		Multivariate^a^	
				HR (95% CI)	*p*‐value^c^	HR (95% CI)	*p*‐value^c^^,^ d
Total	7328	315	0.85				
Age at ART initiation (years)							
≤30	2309	74	0.68	1	<0.001	1	**<0.001**
31 to 40	3110	116	0.71	1.12 (0.83, 1.50)	0.473	1.13 (0.84, 1.53)	0.419
41 to 50	1347	64	0.92	1.40 (0.99, 1.97)	0.056	1.49 (1.05, 2.13)	**0.027**
>50	562	61	2.16	3.40 (2.39, 4.84)	<0.001	3.90 (2.70, 5.62)	**<0.001**
Sex							
Male	5132	249	0.97	1		1	
Female	2196	66	0.59	0.58 (0.44, 0.78)	<0.001	0.70 (0.53, 0.94)	**0.017**
HIV exposure							
Heterosexual contact	4665	214	0.87	1	<0.001	1	0.204
Homosexual contact	1573	42	0.55	0.42 (0.28, 0.63)	<0.001	0.64 (0.42, 0.98)	0.039
Injecting drug use	554	35	1.65	1.69 (1.10, 2.58)	0.016	0.98 (0.60, 1.60)	0.939
Other/unknown	536	24	0.9	0.85 (0.53, 1.34)	0.482	1.02 (0.63, 1.63)	0.946
Hepatitis B co‐infection							
Negative	5036	193	0.75	1		1	
Positive	591	36	1.19	1.53 (1.07, 2.20)	0.019	1.39 (0.96, 2.01)	0.078
Not tested	1701	86	1.06				
Hepatitis C co‐infection							
Negative	4536	172	0.72	1		1	
Positive	786	53	1.61	2.31 (1.64, 3.26)	<0.001	1.90 (1.33, 2.72)	**<0.001**
Not tested	2006	90	0.94				
New TB diagnosis after TAHOD entry							
No	–	276	0.78	1		1	
Yes	–	39	2.94	3.82 (2.68, 5.43)	<0.001	2.50 (1.73, 3.63)	**<0.001**
Viral load (copies/mL)							
<400	–	130	0.49	1	<0.001	1	**0.012**
400 to 999	–	3	0.51	0.97 (0.30, 3.08)	0.958	0.69 (0.21, 2.24)	0.537
1000 to 4999	–	6	0.98	1.76 (0.76, 4.10)	0.189	1.23 (0.53, 2.86)	0.638
≥5000	–	84	2.68	3.89 (2.68, 5.64)	<0.001	1.59 (1.09, 2.34)	**0.017**
Missing	–	92	1.54				
CD4 (cells/μL)							
≤50	–	89	10.39	1	<0.001	1	**<0.001**
51 to 100	–	42	4.47	0.43 (0.30, 0.64)	<0.001	0.42 (0.29, 0.62)	**<0.001**
101 to 200	–	60	1.65	0.17 (0.12, 0.25)	<0.001	0.19 (0.13, 0.28)	**<0.001**
>200	–	115	0.37	0.04 (0.03, 0.06)	<0.001	0.06 (0.04, 0.09)	**<0.001**
Missing	–	9	2.83				
BMI (kg/m^2^)							
<25	–	229	0.93	1		1	
≥25	–	16	0.28	0.33 (0.20, 0.55)	<0.001	0.40 (0.24, 0.68)	**0.001**
Missing	–	70	1.07				
Cotrimoxazole use							
No	–	200	0.66	1		1	
Yes	–	115	1.76	1.91 (1.46, 2.51)	<0.001	0.78 (0.58, 1.03)	0.081
Isoniazid use							
No	–	301	0.84	1		1	
Yes	–	14	1.21	1.71 (0.96, 3.07)	0.070	1.56 (0.86, 2.84)	0.142

^a^Non‐significant covariates were presented in the final model adjusted for the significant covariates; however, they did not form part of the final model; ^b^TB diagnosis, viral load, CD4, BMI, cotrimoxazole and isoniazid use are time‐updated variables; ^c^global *p*‐values for age, VL and CD4 are tests for trend. All other global *p*‐values are tests for heterogeneity excluding missing values; ^d^ *p*‐values in bold represent significant covariates in the final model.

### 3.3 Sensitivity analyses (a) and (b)

Table S2 shows competing risk analysis of factors associated with having presumptive TB diagnosis after cohort entry, with definitive TB analysed as a competing risk (sensitivity analysis (a)). Of the 7355 patients, 227 met the definition of presumptive TB outcome for this sensitivity analysis, which included 102 cases of unreported TB category. The incidence rate was 0.61/100 pys. In the multivariate model, we saw similar risk factors and effect sizes as in Table 1, with hepatitis C co‐infection (subhazard ratio (SHR):1.69, 95% CI (1.08 to 2.65), *p *=* *0.022) and prior TB diagnosis (SHR = 1.37, 95% CI (1.02 to 1.85), *p *=* *0.039) being associated with having presumptive TB. In sensitivity analysis (b), where we analysed factors associated with definitive TB, with presumptive TB as a competing risk, the incidence rate was 0.38/100 pys (Table S3). Due to the small number of events in this analysis, only VL, CD4 and age were associated with definitive TB diagnosis. However, when comparing the results across all three analyses (Table 1, Tables S2 and S3), the effects of each variable were similar, with CTX showing reduction in hazards for TB after adjusting for CD4 cell count.

### 3.4 Sensitivity analyses (c) and (d)

We assessed factors associated with TB diagnosis separately in males and females. Of the 5150 males, 280 (5%) were diagnosed with TB after cohort enrolment, with an incidence rate of 1.08/100 pys (Table S4). Among 2205 females, there were 88 patients (4%) with TB diagnosis, with an incidence rate of 0.78/100 pys (Table S5). In males, being hepatitis C coinfected and having high VL were associated with having TB. Males with high CD4 cell count, BMI above 25 kg/m^2^, been on cART for longer than six months, and receiving CTX had reduced hazards for TB. For females, similar effects were seen; however, hepatitis C co‐infection and CTX were no longer significantly associated with TB.

## 4 Discussion

Our analysis found that cotrimoxazole preventive therapy reduced the hazard for incident TB infection by approximately one‐third in HIV‐infected adult patients, adjusted for HIV viral load, CD4 count, BMI and cART duration. This adds to clinical evidence supporting the potential additive preventive effect of CTX against tuberculosis in HIV‐infected individuals 12, 13. We also found CTX was associated with reduced hazards for TB among those with current CD4 ≤ 50 and 101 to 200 cells/μL.

Previous clinical trials have shown that CTX prophylaxis reduced mortality and hospital admission for septicaemia and enteritis in HIV/TB‐coinfected patients in West Africa, as well as death including tuberculosis and other HIV‐associated conditions in Southeast Asia 8, 9. Such benefits are likely due to a vast array of antibacterial, antifungal, and antiparasitic effects from CTX. In addition, sulphamethoxazole has been found active against Mtb *in vitro* 5, 6, 7. Several observational studies including ours suggested CTX decreased new TB incidence in HIV‐infected individuals, supporting a direct antitubercular effect from CTX 12, 13.

Our findings that higher BMI, greater CD4 cell count, and duration of receiving antiretroviral therapy more than six months were associated with lower incidence of new TB infection were also comparable to other studies 12, 13, 14. The finding that a higher HIV viral load, regardless cART or CD4 cell count was independently associated with an increased risk of new TB infection, is consistent with a previous study in Spain, suggesting that a high HIV viral load in treatment‐naïve patients, in patients with treatment interruption or even in treatment‐experienced patients with a failing antiretroviral regimen may be linked to an increased occurrence of active TB 20.

The reversal of the HRs for CTX once CD4 cell count was adjusted for reflects the confounding of CTX by CD4 cell count. CTX is normally prescribed as primary prophylaxis in patients with CD4 < 200 cells/μL 1, who are more likely to have poorer treatment outcomes. When CTX was analysed in the univariate analysis, the increased hazard for incident TB in those receiving CTX simply reflected the underlying confounding of increased TB in those with low CD4 counts. Once the confounding CD4 levels were controlled for in the multivariate analysis, that is once we compared the effects of CTX in patients within the same CD4 category, it was evident that CTX reduced the hazard for TB diagnosis.

Many studies have shown that CTX preventive therapy reduces mortality in HIV‐infected patients 21, 22. A previous TAHOD study showed greatest absolute survival benefit from PJP prophylaxis, predominantly with CTX, in patients with a CD4 count less than 50 cells/μL 23. While this study showed improved survival in those using CTX, the effect was only statistically significant in people with current CD4 101 to 200 cells/μL, possibly due to attenuation of the benefit by including only patients who had initiated cART in the current analysis.

We did not find differences in survival time according to isoniazid use in the multivariate analyses, which was likely due to the small number of patients that had actually received isoniazid preventive therapy (IPT) in our cohort. This finding might also reflect the fact that IPT is not delivered uniformly by physicians in concordance with WHO or local guidelines in our region 24.

While our study analysed data on a substantial number of patients from a prospective cohort in a region where TB burden is high, several important limitations are noted. Firstly, not all new TB cases were laboratory‐confirmed with positive culture results since we used both definite and presumptive definitions for TB diagnosis. While this helps to avoid underascertainment of TB cases, we may have missed other patients who were unknown to be receiving care outside of the HIV clinical setting. We also performed sensitivity analyses, and the effects of each variable were similar in new cases with both definite and presumptive TB diagnosis. Tuberculin skin testing (TST) results were not recorded in our cohort thus patients with possible latent TB infection were not excluded from the analyses. Although the prescription of prophylactic CTX was documented, we did not specifically assess adherence to CTX or precise dosage of CTX in our cohort sites. A recent pharmacokinetic/ pharmacodynamic study showed that the serum level of sulphamethoxazole is comparable with other drugs with anti‐TB activity, like pyrazinamide, at a standard prophylactic dose of 960 mg CTX once daily, as recommended by WHO 25. As we have considered TB diagnosis within 60 days of initiation of CTX to be considered as not exposed to CTX, this may accentuate the protective effect of CTX if TB cases were diagnosed soon after cART initiation. Lastly, the susceptibility test results to CTX or other antimycobacterial agents for the microbiologic isolates were not collected in our TAHOD database. Nevertheless, other studies have shown that the minimal inhibitory concentration of sulphamethoxazole is not significantly different in patients infected with MDR‐TB or drug‐susceptible TB and that resistance to sulphamethoxazole was not frequent in TB/HIV‐coinfected patients taking CTX prophylaxis 26, 27.

## 5 Conclusions

Our study found that cotrimoxazole preventive therapy was associated with a reduction in the hazard for incident TB infection among Asian patients in our cohort, adding to existing clinical evidence supporting the use of CTX in HIV‐infected patients for broader prevention purposes.

## Competing Interests

The authors have none to declare.

## Authors’ Contributions

SWWK, KJ, MKP, AW, RC, SK, AA, PSL, NK, CDD, TPM, KVN, AK, FJZ, MPL, JYC, JT, SK, BLHS, OTN, WR and WWW were involved in data collection. SWWK, AJ, JM and WWW were involved in data analysis. SWWK, AJ, KJ, MKP, AW, RC, JM and WWW were involved in data interpretation and presentation of the results. All authors have read and approved the final manuscript.

## Supporting information


**Table S1.** Factors associated with TB diagnosis, stratified by both CD4 category and site
**Table S2.** Factors associated with Presumptive TB diagnosis
**Table S3.** Factors associated with Definitive TB diagnosis
**Table S4.** Factors associated with TB diagnosis in males
**Table S5.** Factors associated with TB diagnosis in females

## Appendix 1 The TREAT Asia HIV Observational Database

### 1.1

PS Ly (National Center for HIV/AIDS, Dermatology & STDs, Phnom Penh, Cambodia; TAHOD Steering Committee member); V Khol (National Center for HIV/AIDS, Dermatology & STDs, Phnom Penh, Cambodia); FJ Zhang (Beijing Ditan Hospital, Capital Medical University, Beijing, China; TAHOD Steering Committee member; Steering Committee Chair); HX Zhao (Beijing Ditan Hospital, Capital Medical University, Beijing, China); N Han (Beijing Ditan Hospital, Capital Medical University, Beijing, China); MP Lee (Queen Elizabeth Hospital, Hong Kong SAR; TAHOD Steering Committee member); PCK Li (Queen Elizabeth Hospital, Hong Kong SAR); W Lam (Queen Elizabeth Hospital, Hong Kong SAR); YT Chan (Queen Elizabeth Hospital, Hong Kong SAR); N Kumarasamy (Chennai Antiviral Research and Treatment Clinical Research Site (CART CRS); YRGCARE Medical Centre, VHS, Chennai, India; TAHOD Steering Committee member); S Saghayam (Chennai Antiviral Research and Treatment Clinical Research Site (CART CRS), YRGCARE Medical Centre, VHS, Chennai, India); C Ezhilarasi (Chennai Antiviral Research and Treatment Clinical Research Site (CART CRS), YRGCARE Medical Centre, VHS, Chennai, India); S Pujari (Institute of Infectious Diseases, Pune, India; TAHOD Steering Committee member); K Joshi (Institute of Infectious Diseases, Pune, India); S Gaikwad (Institute of Infectious Diseases, Pune, India); A Chitalikar (Institute of Infectious Diseases, Pune, India); S Sangle (BJ Government Medical College and Sassoon General Hospital, Pune, India; TAHOD Steering Committee member); V Mave (BJ Government Medical College and Sassoon General Hospital, Pune, India); I Marbaniang (BJ Government Medical College and Sassoon General Hospital, Pune, India) TP Merati (Faculty of Medicine Udayana University & Sanglah Hospital, Bali, Indonesia; TAHOD Steering Committee member); DN Wirawan (Faculty of Medicine Udayana University & Sanglah Hospital, Bali, Indonesia); F Yuliana (Faculty of Medicine Udayana University & Sanglah Hospital, Bali, Indonesia); E Yunihastuti (Faculty of Medicine Universitas Indonesia ‐ Dr. Cipto Mangunkusumo General Hospital, Jakarta, Indonesia; TAHOD Steering Committee member); D Imran (Faculty of Medicine Universitas Indonesia ‐ Dr. Cipto Mangunkusumo General Hospital, Jakarta, Indonesia); A Widhani (Faculty of Medicine Universitas Indonesia ‐ Dr. Cipto Mangunkusumo General Hospital, Jakarta, Indonesia); J Tanuma (National Center for Global Health and Medicine, Tokyo, Japan; TAHOD Steering Committee member); S Oka (National Center for Global Health and Medicine, Tokyo, Japan); T Nishijima (National Center for Global Health and Medicine, Tokyo, Japan); JY Choi (Division of Infectious Diseases, Department of Internal Medicine, Yonsei University College of Medicine, Seoul, South Korea; TAHOD Steering Committee member); Na S (Division of Infectious Diseases, Department of Internal Medicine, Yonsei University College of Medicine, Seoul, South Korea); JM Kim (Division of Infectious Diseases, Department of Internal Medicine, Yonsei University College of Medicine, Seoul, South Korea); BLH Sim (Hospital Sungai Buloh, Sungai Buloh, Malaysia; TAHOD Steering Committee member); YM Gani (Hospital Sungai Buloh, Sungai Buloh, Malaysia); NB Rudi (Hospital Sungai Buloh, Sungai Buloh, Malaysia); A Kamarulzaman (University Malaya Medical Centre, Kuala Lumpur, Malaysia; TAHOD Steering Committee member); SF Syed Omar (University Malaya Medical Centre, Kuala Lumpur, Malaysia); S Ponnampalavanar (University Malaya Medical Centre, Kuala Lumpur, Malaysia); I Azwa (University Malaya Medical Centre, Kuala Lumpur, Malaysia); R Ditangco (Research Institute for Tropical Medicine, Muntinlupa City, Philippines; TAHOD Steering Committee member); MK Pasayan (Research Institute for Tropical Medicine, Muntinlupa City, Philippines); ML Mationg (Research Institute for Tropical Medicine, Muntinlupa City, Philippines); WW Wong (Taipei Veterans General Hospital, Taipei, Taiwan; TAHOD Steering Committee member); SWW Ku (Taipei Veterans General Hospital, Taipei, Taiwan); PC Wu (Taipei Veterans General Hospital, Taipei, Taiwan); OT Ng (Tan Tock Seng Hospital, Singapore; TAHOD Steering Committee member; co‐Chair); PL Lim (Tan Tock Seng Hospital, Singapore); LS Lee (Tan Tock Seng Hospital, Singapore); Z Ferdous (Tan Tock Seng Hospital, Singapore); A Avihingsanon (HIV‐NAT/Thai Red Cross AIDS Research Centre, Bangkok, Thailand; TAHOD Steering Committee member); S Gatechompol (HIV‐NAT/Thai Red Cross AIDS Research Centre, Bangkok, Thailand); P Phanuphak (HIV‐NAT/Thai Red Cross AIDS Research Centre, Bangkok, Thailand); C Phadungphon (HIV‐NAT/Thai Red Cross AIDS Research Centre, Bangkok, Thailand); S Kiertiburanakul (Faculty of Medicine Ramathibodi Hospital, Mahidol University, Bangkok, Thailand; TAHOD Steering Committee member); A Phuphuakrat (Faculty of Medicine Ramathibodi Hospital, Mahidol University, Bangkok, Thailand); L Chumla (Faculty of Medicine Ramathibodi Hospital, Mahidol University, Bangkok, Thailand); N Sanmeema (Faculty of Medicine Ramathibodi Hospital, Mahidol University, Bangkok, Thailand); R Chaiwarith (Research Institute for Health Sciences, Chiang Mai, Thailand; TAHOD Steering Committee member); T Sirisanthana (Research Institute for Health Sciences, Chiang Mai, Thailand); W Kotarathititum (Research Institute for Health Sciences, Chiang Mai, Thailand); J Praparattanapan (Research Institute for Health Sciences, Chiang Mai, Thailand); S Khusuwan (Chiangrai Prachanukroh Hospital, Chiang Rai, Thailand; TAHOD Steering Committee member); P Kantipong (Chiangrai Prachanukroh Hospital, Chiang Rai, Thailand); P Kambua (Chiangrai Prachanukroh Hospital, Chiang Rai, Thailand); W Ratanasuwan (Faculty of Medicine, Siriraj Hospital, Mahidol University, Bangkok, Thailand; TAHOD Steering Committee member); R Sriondee (Faculty of Medicine, Siriraj Hospital, Mahidol University, Bangkok, Thailand); KV Nguyen (National Hospital for Tropical Diseases, Hanoi, Vietnam; TAHOD Steering Committee member); HV Bui (National Hospital for Tropical Diseases, Hanoi, Vietnam); DTH Nguyen (National Hospital for Tropical Diseases, Hanoi, Vietnam); DT Nguyen (National Hospital for Tropical Diseases, Hanoi, Vietnam); CD Do (Bach Mai Hospital, Hanoi, Vietnam; TAHOD Steering Committee member); AV Ngo (Bach Mai Hospital, Hanoi, Vietnam); LT Nguyen (Bach Mai Hospital, Hanoi, Vietnam); AH Sohn (TREAT Asia, amfAR ‐ The Foundation for AIDS Research, Bangkok, Thailand; TAHOD Steering Committee member); JL Ross (TREAT Asia, amfAR ‐ The Foundation for AIDS Research, Bangkok, Thailand; TAHOD Steering Committee member); B Petersen (TREAT Asia, amfAR – The Foundation for AIDS Research, Bangkok, Thailand); DA Cooper (The Kirby Institute, UNSW Sydney, NSW, Australia); MG Law (The Kirby Institute, UNSW Sydney, NSW, Australia; TAHOD Steering Committee member); A Jiamsakul (The Kirby Institute, UNSW Sydney, NSW, Australia; TAHOD Steering Committee member); D Rupasinghe (The Kirby Institute, UNSW Sydney, NSW, Australia).
