# Supplementary material for: Cotrimoxazole prophylaxis decreases tuberculosis risk among Asian patients with HIV
Source: J Int AIDS Soc. 2019 Mar 29;22(3):e25264. doi: 10.1002/jia2.25264 (PMC6439318; doi:10.1002/jia2.25264)
Supplement: Supplementary file 1 — Table S1. Factors associated with TB diagnosis, stratified by both CD4 category and site Table S2. Factors associated with Presumptive TB diagnosis Table S3. Factors associated with Definitive TB diagnosis Table S4. Factors associated with TB diagnosis in males Table S5. Factors associated with TB diagnosis in females [file JIA2-22-e25264-s001.docx]

Supplementary Table 1: Factors associated with TB diagnosis, stratified by CD4 categories and site

|  |  |  |  | **Univariate** | | | **Multivariate^b^** | | |
| --- | --- | --- | --- | --- | --- | --- | --- | --- | --- |
|  | **No of patients^d^** | **No of TB diagnosis** | **Rate (/100pys)** | **HR** | **95% CI** | **p-value^a^** | **HR** | **95% CI** | **p-value^a,c^** |
| **Total** | **7355** | **368** | **0.99** |  |  |  |  |  |  |
| **Age at TAHOD entry (years)** |  |  |  |  |  | 0.574 |  |  | 0.111 |
| ≤30 | 1982 | 100 | 1.07 | 1 |  |  | 1 |  |  |
| 31-40 | 3205 | 175 | 1.05 | 1.18 | (0.91, 1.53) | 0.225 | 1.29 | (0.99, 1.68) | 0.059 |
| 41-50 | 1513 | 73 | 0.94 | 1.29 | (0.94, 1.78) | 0.115 | 1.48 | (1.07, 2.05) | 0.018 |
| >50 | 655 | 20 | 0.59 | 0.87 | (0.53, 1.44) | 0.599 | 1.08 | (0.65, 1.79) | 0.767 |
| **Sex** |  |  |  |  |  |  |  |  |  |
| Male | 5150 | 280 | 1.08 | 1 |  |  | 1 |  |  |
| Female | 2205 | 88 | 0.78 | 0.87 | (0.68, 1.13) | 0.304 | 0.81 | (0.63, 1.05) | 0.109 |
| **HIV Exposure** |  |  |  |  |  | 0.749 |  |  | 0.589 |
| Heterosexual contact | 4682 | 253 | 1.03 | 1 |  |  | 1 |  |  |
| Homosexual contact | 1580 | 50 | 0.63 | 0.90 | (0.61, 1.33) | 0.594 | 0.89 | (0.60, 1.31) | 0.548 |
| Injecting drug use | 555 | 41 | 1.99 | 1.22 | (0.79, 1.90) | 0.374 | 1.29 | (0.82, 2.02) | 0.267 |
| Other/Unknown | 538 | 24 | 0.88 | 0.93 | (0.58, 1.50) | 0.765 | 0.88 | (0.55, 1.43) | 0.615 |
| **Hepatitis B co-infection** |  |  |  |  |  |  |  |  |  |
| Negative | 5050 | 234 | 0.90 | 1 |  |  | 1 |  |  |
| Positive | 594 | 26 | 0.86 | 0.93 | (0.61, 1.44) | 0.758 | 1.02 | (0.66, 1.58) | 0.919 |
| Not tested | 1711 | 108 | 1.33 |  |  |  |  |  |  |
| **Hepatitis C co-infection** |  |  |  |  |  |  |  |  |  |
| Negative | 4544 | 186 | 0.77 | 1 |  |  | 1 |  |  |
| Positive | 787 | 42 | 1.27 | 1.36 | (0.90, 2.06) | 0.146 | 1.40 | (0.93, 2.12) | 0.111 |
| Not tested | 2024 | 140 | 1.45 |  |  |  |  |  |  |
| **Prior TB** |  |  |  |  |  |  |  |  |  |
| No | 5856 | 239 | 0.80 | 1 |  |  | 1 |  |  |
| Yes | 1499 | 129 | 1.77 | 1.11 | (0.87, 1.41) | 0.400 | 1.23 | (0.96, 1.58) | 0.094 |
| **Viral Load (copies/mL)** |  |  |  |  |  | <0.001 |  |  | **<0.001** |
| <400 | ~ | 76 | 0.30 | 1 |  |  | 1 |  |  |
| 400-999 | ~ | 3 | 0.55 | 0.99 | (0.31, 3.21) | 0.990 | 0.80 | (0.25, 2.59) | 0.708 |
| 1000-4999 | ~ | 9 | 1.24 | 2.59 | (1.24, 5.39) | 0.011 | **2.03** | **(0.97, 4.25)** | **0.060** |
| ≥5000 | ~ | 100 | 2.93 | 2.63 | (1.79, 3.86) | <0.001 | **1.91** | **(1.28, 2.83)** | **0.001** |
| Not reported | ~ | 180 | 2.47 |  |  |  |  |  |  |
| **BMI (kg/m^2^)** |  |  |  |  |  |  |  |  |  |
| <25 | ~ | 265 | 1.09 | 1 |  |  | 1 |  |  |
| ≥25 | ~ | 27 | 0.46 | 0.50 | (0.33, 0.75) | 0.001 | **0.51** | **(0.34, 0.76)** | **0.001** |
| Not reported | ~ | 76 | 1.09 |  |  |  |  |  |  |
| **cART duration** |  |  |  |  |  | <0.001 |  |  | **<0.001** |
| Prior to cART initiation | ~ | 53 | 3.20 | 1 |  |  | 1 |  |  |
| <6 months | ~ | 84 | 5.43 | 0.86 | (0.55, 1.34) | 0.513 | 1.03 | (0.66, 1.62) | 0.882 |
| 6-12 months | ~ | 29 | 1.45 | 0.37 | (0.21, 0.63) | <0.001 | **0.47** | **(0.27, 0.81)** | **0.007** |
| >12 months | ~ | 202 | 0.63 | 0.27 | (0.19, 0.39) | <0.001 | **0.36** | **(0.25, 0.52)** | **<0.001** |
| **Cotrimoxazole use** |  |  |  |  |  |  |  |  |  |
| No | ~ | 231 | 0.75 | 1 |  |  | 1 |  |  |
| Yes | ~ | 137 | 2.13 | 0.72 | (0.54, 0.95) | 0.020 | **0.67** | **(0.51, 0.89)** | **0.005** |
| **Isoniazid use** |  |  |  |  |  |  |  |  |  |
| No | ~ | 353 | 0.98 | 1 |  |  | 1 |  |  |
| Yes | ~ | 15 | 1.31 | 1.08 | (0.62, 1.90) | 0.778 | 1.23 | (0.69, 2.17) | 0.488 |
| ^a^ Global p-values for age, and VL are tests for trend. All other global p-values are tests for heterogeneity excluding missing values.  ^b^ Non-significant covariates were presented in the final model adjusted for the significant covariates, however they did not form part of the final model.  ^c^ P-values in bold represent significant covariates in the final model.  ^d^ ~ Viral load, BMI, cART duration, cotrimoxazole and isoniazid use are time-updated variables. | | | | | | | | | |

Supplementary Table 2: Factors associated with presumptive TB diagnosis

|  |  |  |  | **Univariate** | | | **Multivariate^b^** | | |
| --- | --- | --- | --- | --- | --- | --- | --- | --- | --- |
|  | **No of patients^d^** | **No of TB diagnosis** | **Rate (/100pys)** | **SHR** | **95% CI** | **p-value^a^** | **SHR** | **95% CI** | **p-value^a,c^** |
| **Total** | **7355** | **227** | **0.61** |  |  |  |  |  |  |
| **Age at TAHOD entry (years)** |  |  |  |  |  | 0.649 |  |  | 0.437 |
| ≤30 | 1982 | 73 | 0.78 | 1 |  |  | 1 |  |  |
| 31-40 | 3205 | 94 | 0.56 | 0.86 | (0.63, 1.18) | 0.358 | 0.91 | (0.67, 1.25) | 0.564 |
| 41-50 | 1513 | 47 | 0.60 | 1.01 | (0.70, 1.48) | 0.938 | 1.23 | (0.83, 1.81) | 0.297 |
| >50 | 655 | 13 | 0.38 | 0.78 | (0.43, 1.41) | 0.412 | 0.93 | (0.51, 1.71) | 0.821 |
| **Sex** |  |  |  |  |  |  |  |  |  |
| Male | 5150 | 171 | 0.66 | 1 |  |  | 1 |  |  |
| Female | 2205 | 56 | 0.49 | 0.81 | (0.59, 1.11) | 0.185 | 0.85 | (0.62, 1.17) | 0.312 |
| **HIV Exposure** |  |  |  |  |  | 0.060 |  |  | 0.418 |
| Heterosexual contact | 4682 | 148 | 0.60 | 1 |  |  | 1 |  |  |
| Homosexual contact | 1580 | 31 | 0.39 | 0.73 | (0.46, 1.17) | 0.189 | 0.87 | (0.54, 1.40) | 0.565 |
| Injecting drug use | 555 | 30 | 1.46 | 1.82 | (1.09, 3.06) | 0.023 | 1.49 | (0.90, 2.49) | 0.124 |
| Other/Unknown | 538 | 18 | 0.66 | 0.94 | (0.55, 1.59) | 0.812 | 1.03 | (0.60, 1.75) | 0.921 |
| **Hepatitis B co-infection** |  |  |  |  |  |  |  |  |  |
| Negative | 5050 | 139 | 0.53 | 1 |  |  | 1 |  |  |
| Positive | 594 | 13 | 0.43 | 0.89 | (0.50, 1.59) | 0.695 | 0.91 | (0.51, 1.66) | 0.769 |
| Not tested | 1711 | 75 | 0.92 |  |  |  |  |  |  |
| **Hepatitis C co-infection** |  |  |  |  |  |  |  |  |  |
| Negative | 4544 | 113 | 0.47 | 1 |  |  | 1 |  |  |
| Positive | 787 | 30 | 0.91 | 1.84 | (1.18, 2.86) | 0.007 | **1.69** | **(1.08, 2.65)** | **0.022** |
| Not tested | 2024 | 84 | 0.87 |  |  |  |  |  |  |
| **Prior TB** |  |  |  |  |  |  |  |  |  |
| No | 5856 | 145 | 0.48 | 1 |  |  | 1 |  |  |
| Yes | 1499 | 82 | 1.13 | 1.50 | (1.12, 2.01) | 0.006 | **1.37** | **(1.02, 1.85)** | **0.039** |
| **Viral Load (copies/mL)** |  |  |  |  |  | <0.001 |  |  | **0.014** |
| <400 | ~ | 38 | 0.15 | 1 |  |  | 1 |  |  |
| 400-999 | ~ | 3 | 0.55 | 2.46 | (0.77, 7.82) | 0.128 | 1.52 | (0.46, 5.00) | 0.495 |
| 1000-4999 | ~ | 6 | 0.83 | 3.99 | (1.64, 9.71) | 0.002 | **2.55** | **(1.05, 6.23)** | **0.039** |
| ≥5000 | ~ | 59 | 1.73 | 5.65 | (3.59, 8.89) | <0.001 | **2.15** | **(1.32, 3.49)** | **0.002** |
| Not reported | ~ | 121 | 1.66 |  |  |  |  |  |  |
| **CD4 (cells/uL)** |  |  |  |  |  | <0.001 |  |  | **<0.001** |
| ≤50 | ~ | 58 | 7.37 | 1 |  |  | 1 |  |  |
| 51-100 | ~ | 16 | 1.85 | 0.29 | (0.16, 0.51) | <0.001 | **0.34** | **(0.19, 0.60)** | **<0.001** |
| 101-200 | ~ | 49 | 1.38 | 0.28 | (0.19, 0.43) | <0.001 | **0.38** | **(0.25, 0.57)** | **<0.001** |
| >200 | ~ | 88 | 0.28 | 0.08 | (0.05, 0.12) | <0.001 | **0.11** | **(0.06, 0.18)** | **<0.001** |
| Not reported | ~ | 16 | 2.86 |  |  |  |  |  |  |
| **BMI (kg/m^2^)** |  |  |  |  |  |  |  |  |  |
| <25 | ~ | 157 | 0.64 | 1 |  |  | 1 |  |  |
| ≥25 | ~ | 13 | 0.22 | 0.33 | (0.19, 0.58) | <0.001 | **0.40** | **(0.23, 0.71)** | **0.002** |
| Not reported | ~ | 57 | 0.82 |  |  |  |  |  |  |
| **cART duration** |  |  |  |  |  | <0.001 |  |  | **<0.001** |
| Prior to cART initiation | ~ | 34 | 2.05 | 1 |  |  | 1 |  |  |
| <6 months | ~ | 61 | 3.94 | 1.59 | (0.96, 2.65) | 0.074 | 0.92 | (0.51, 1.67) | 0.795 |
| 6-12 months | ~ | 19 | 0.95 | 0.43 | (0.24, 0.76) | 0.004 | **0.38** | **(0.20, 0.72)** | **0.003** |
| >12 months | ~ | 113 | 0.35 | 0.28 | (0.19, 0.42) | <0.001 | **0.34** | **(0.22, 0.53)** | **<0.001** |
| **Cotrimoxazole use** |  |  |  |  |  |  |  |  |  |
| No | ~ | 145 | 0.47 | 1 |  |  | 1 |  |  |
| Yes | ~ | 82 | 1.28 | 1.46 | (1.11, 1.92) | 0.008 | **0.64** | **(0.46, 0.89)** | **0.008** |
| **Isoniazid use** |  |  |  |  |  |  |  |  |  |
| No | ~ | 215 | 0.60 | 1 |  |  | 1 |  |  |
| Yes | ~ | 12 | 1.05 | 1.07 | (0.58, 1.99) | 0.826 | 0.99 | (0.53, 1.84) | 0.968 |
| SHR – Sub-hazard ratio  ^a^ Global p-values are tests for heterogeneity excluding missing values. Test for trend was not performed in the competing risk model.  ^b^ Non-significant covariates were presented in the final model adjusted for the significant covariates, however they did not form part of the final model.  ^c^ P-values in bold represent significant covariates in the final model.  ^d^ ~ Viral load, CD4, BMI, cART duration, cotrimoxazole and isoniazid use are time-updated variables. | | | | | | | | | |

Supplementary Table 3: Factors associated with definitive TB diagnosis

|  |  |  |  | **Univariate** | | | **Multivariate^b^** | | |
| --- | --- | --- | --- | --- | --- | --- | --- | --- | --- |
|  | **No of patients^d^** | **No of TB diagnosis** | **Rate (/100pys)** | **SHR** | **95% CI** | **p-value^a^** | **SHR** | **95% CI** | **p-value^a,c^** |
| **Total** | **7355** | **141** | **0.38** |  |  |  |  |  |  |
| **Age at TAHOD entry (years)** |  |  |  |  |  | 0.030 |  |  | **0.006** |
| ≤30 | 1982 | 27 | 0.29 | 1 |  |  | 1 |  |  |
| 31-40 | 3205 | 81 | 0.48 | 1.78 | (1.16, 2.73) | 0.008 | **2.08** | **(1.35, 3.19)** | **0.001** |
| 41-50 | 1513 | 26 | 0.33 | 1.37 | (0.80, 2.36) | 0.254 | **1.76** | **(1.01, 3.08)** | **0.047** |
| >50 | 655 | 7 | 0.21 | 0.91 | (0.39, 2.12) | 0.831 | 1.07 | (0.45, 2.52) | 0.875 |
| **Sex** |  |  |  |  |  |  |  |  |  |
| Male | 5150 | 109 | 0.42 | 1 |  |  | 1 |  |  |
| Female | 2205 | 32 | 0.28 | 0.68 | (0.45, 1.02) | 0.064 | 0.84 | (0.55, 1.28) | 0.416 |
| **HIV Exposure** |  |  |  |  |  | 0.960 |  |  | 0.969 |
| Heterosexual contact | 4682 | 105 | 0.43 | 1 |  |  | 1 |  |  |
| Homosexual contact | 1580 | 19 | 0.24 | 0.86 | (0.43, 1.69) | 0.658 | 0.96 | (0.46, 1.99) | 0.910 |
| Injecting drug use | 555 | 11 | 0.53 | 1.04 | (0.42, 2.58) | 0.925 | 0.91 | (0.37, 2.23) | 0.844 |
| Other/Unknown | 538 | 6 | 0.22 | 0.82 | (0.31, 2.14) | 0.680 | 0.81 | (0.33, 2.00) | 0.648 |
| **Hepatitis B co-infection** |  |  |  |  |  |  |  |  |  |
| Negative | 5050 | 95 | 0.36 | 1 |  |  | 1 |  |  |
| Positive | 594 | 13 | 0.43 | 1.50 | (0.84, 2.70) | 0.170 | 1.25 | (0.70, 2.24) | 0.455 |
| Not tested | 1711 | 33 | 0.41 |  |  |  |  |  |  |
| **Hepatitis C co-infection** |  |  |  |  |  |  |  |  |  |
| Negative | 4544 | 73 | 0.30 | 1 |  |  | 1 |  |  |
| Positive | 787 | 12 | 0.36 | 0.89 | (0.42, 1.90) | 0.770 | 0.76 | (0.35, 1.65) | 0.491 |
| Not tested | 2024 | 56 | 0.58 |  |  |  |  |  |  |
| **Prior TB** |  |  |  |  |  |  |  |  |  |
| No | 5856 | 94 | 0.31 | 1 |  |  | 1 |  |  |
| Yes | 1499 | 47 | 0.64 | 1.09 | (0.76, 1.57) | 0.639 | 0.87 | (0.59, 1.28) | 0.481 |
| **Viral Load (copies/mL)** |  |  |  |  |  | <0.001 |  |  | **<0.001** |
| <400 | ~ | 38 | 0.15 | 1 |  |  | 1 |  |  |
| 400-999 | ~ | 0 | 0.00 | N/A | N/A | N/A | N/A | N/A | N/A |
| 1000-4999 | ~ | 3 | 0.41 | 2.58 | (0.78, 8.55) | 0.120 | 2.43 | (0.73, 8.12) | 0.148 |
| ≥5000 | ~ | 41 | 1.20 | 6.49 | (4.06, 10.37) | <0.001 | **4.42** | **(2.70, 7.21)** | **<0.001** |
| Not reported | ~ | 59 | 0.81 |  |  |  |  |  |  |
| **CD4 (cells/uL)** |  |  |  |  |  | <0.001 |  |  | **<0.001** |
| ≤50 | ~ | 20 | 2.54 | 1 |  |  | 1 |  |  |
| 51-100 | ~ | 19 | 2.20 | 1.10 | (0.57, 2.13) | 0.785 | 1.27 | (0.65, 2.47) | 0.485 |
| 101-200 | ~ | 28 | 0.79 | 0.48 | (0.26, 0.90) | 0.021 | 0.65 | (0.34, 1.22) | 0.176 |
| >200 | ~ | 70 | 0.22 | 0.14 | (0.08, 0.24) | <0.001 | **0.23** | **(0.13, 0.41)** | **<0.001** |
| Not reported | ~ | 4 | 0.72 |  |  |  |  |  |  |
| **BMI (kg/m^2^)** |  |  |  |  |  |  |  |  |  |
| <25 | ~ | 108 | 0.44 | 1 |  |  | 1 |  |  |
| ≥25 | ~ | 14 | 0.24 | 0.49 | (0.28, 0.86) | 0.013 | 0.60 | (0.34, 1.07) | 0.083 |
| Not reported | ~ | 19 | 0.27 |  |  |  |  |  |  |
| **cART duration** |  |  |  |  |  | <0.001 |  |  | 0.285 |
| Prior to cART initiation | ~ | 19 | 1.15 | 1 |  |  | 1 |  |  |
| <6 months | ~ | 23 | 1.49 | 1.64 | (0.88, 3.05) | 0.119 | 0.98 | (0.48, 2.00) | 0.959 |
| 6-12 months | ~ | 10 | 0.50 | 0.63 | (0.29, 1.37) | 0.241 | 0.63 | (0.27, 1.47) | 0.288 |
| >12 months | ~ | 89 | 0.28 | 0.41 | (0.24, 0.69) | 0.001 | 0.64 | (0.37, 1.13) | 0.123 |
| **Cotrimoxazole use** |  |  |  |  |  |  |  |  |  |
| No | ~ | 86 | 0.28 | 1 |  |  | 1 |  |  |
| Yes | ~ | 55 | 0.86 | 1.92 | (1.30, 2.82) | 0.001 | 0.79 | (0.50, 1.24) | 0.306 |
| **Isoniazid use** |  |  |  |  |  |  |  |  |  |
| No | ~ | 138 | 0.38 | 1 |  |  | 1 |  |  |
| Yes | ~ | 3 | 0.26 | 1.21 | (0.33, 4.48) | 0.775 | 1.06 | (0.31, 3.61) | 0.927 |
| SHR – Sub-hazard ratio  ^a^ Global p-values are tests for heterogeneity excluding missing values. Test for trend was not performed in the competing risk model.  ^b^ Non-significant covariates were presented in the final model adjusted for the significant covariates, however they did not form part of the final model.  ^c^ P-values in bold represent significant covariates in the final model.  ^d^ ~ Viral load, CD4, BMI, cART duration, cotrimoxazole and isoniazid use are time-updated variables. | | | | | | | | | |

Supplementary Table 4: Factors associated with TB diagnosis in males

|  |  |  |  | **Univariate** | | | **Multivariate^b^** | | |
| --- | --- | --- | --- | --- | --- | --- | --- | --- | --- |
|  | **No of patients^d^** | **No of TB diagnosis** | **Rate (/100pys)** | **HR** | **95% CI** | **p-value^a^** | **HR** | **95% CI** | **p-value^a,c^** |
| **Total** | **5150** | **280** | **1.08** |  |  |  |  |  |  |
| **Age at TAHOD entry (years)** |  |  |  |  |  | 0.030 |  |  | **0.006** |
| ≤30 | 1982 | 27 | 0.29 | 1 |  |  | 1 |  |  |
| 31-40 | 3205 | 81 | 0.48 | 1.78 | (1.16, 2.73) | 0.008 | **2.08** | **(1.35, 3.19)** | **0.001** |
| 41-50 | 1513 | 26 | 0.33 | 1.37 | (0.80, 2.36) | 0.254 | **1.76** | **(1.01, 3.08)** | **0.047** |
| >50 | 655 | 7 | 0.21 | 0.91 | (0.39, 2.12) | 0.831 | 1.07 | (0.45, 2.52) | 0.875 |
| **HIV Exposure** |  |  |  |  |  | 0.088 |  |  | 0.740 |
| Heterosexual contact | 2664 | 171 | 1.21 | 1 |  |  | 1 |  |  |
| Homosexual contact | 1568 | 50 | 0.64 | 0.71 | (0.47, 1.07) | 0.105 | 0.82 | (0.54, 1.24) | 0.344 |
| Injecting drug use | 518 | 40 | 2.10 | 1.53 | (0.93, 2.52) | 0.091 | 1.12 | (0.64, 1.97) | 0.685 |
| Other/Unknown | 400 | 19 | 0.95 | 0.80 | (0.47, 1.36) | 0.408 | 0.86 | (0.51, 1.45) | 0.571 |
| **Hepatitis B co-infection** |  |  |  |  |  |  |  |  |  |
| Negative | 3494 | 176 | 0.99 | 1 |  |  | 1 |  |  |
| Positive | 466 | 22 | 0.91 | 1.07 | (0.69, 1.68) | 0.755 | 1.05 | (0.67, 1.65) | 0.825 |
| Not tested | 1190 | 82 | 1.44 |  |  |  |  |  |  |
| **Hepatitis C co-infection** |  |  |  |  |  |  |  |  |  |
| Negative | 3108 | 132 | 0.80 | 1 |  |  | 1 |  |  |
| Positive | 675 | 41 | 1.51 | 1.62 | (1.04, 2.51) | 0.032 | **1.60** | **(1.03, 2.51)** | **0.038** |
| Not tested | 1367 | 107 | 1.63 |  |  |  |  |  |  |
| **Prior TB** |  |  |  |  |  |  |  |  |  |
| No | 3968 | 172 | 0.85 | 1 |  |  | 1 |  |  |
| Yes | 1182 | 108 | 1.89 | 1.32 | (1.01, 1.71) | 0.042 | 1.22 | (0.94, 1.59) | 0.142 |
| **Viral Load (copies/mL)** |  |  |  |  |  | <0.001 |  |  | **<0.001** |
| <400 | ~ | 59 | 0.33 | 1 |  |  | 1 |  |  |
| 400-999 | ~ | 1 | 0.26 | 0.56 | (0.08, 4.11) | 0.573 | 0.38 | (0.05, 2.78) | 0.342 |
| 1000-4999 | ~ | 9 | 1.77 | 4.01 | (1.94, 8.26) | <0.001 | **2.85** | **(1.36, 5.95)** | **0.005** |
| ≥5000 | ~ | 80 | 3.28 | 5.07 | (3.42, 7.53) | <0.001 | **2.32** | **(1.51, 3.56)** | **<0.001** |
| Not reported | ~ | 131 | 2.73 |  |  |  |  |  |  |
| **CD4 (cells/uL)** |  |  |  |  |  | <0.001 |  |  | **<0.001** |
| ≤50 | ~ | 64 | 10.83 | 1 |  |  | 1 |  |  |
| 51-100 | ~ | 30 | 4.68 | 0.50 | (0.32, 0.78) | 0.002 | **0.57** | **(0.36, 0.89)** | **0.013** |
| 101-200 | ~ | 58 | 2.23 | 0.31 | (0.21, 0.45) | <0.001 | **0.39** | **(0.26, 0.57)** | **<0.001** |
| >200 | ~ | 114 | 0.53 | 0.08 | (0.06, 0.12) | <0.001 | **0.10** | **(0.07, 0.14)** | **<0.001** |
| Not reported | ~ | 14 | 3.60 |  |  |  |  |  |  |
| **BMI (kg/m^2^)** |  |  |  |  |  |  |  |  |  |
| <25 | ~ | 202 | 1.26 | 1 |  |  | 1 |  |  |
| ≥25 | ~ | 22 | 0.51 | 0.41 | (0.27, 0.64) | <0.001 | **0.48** | **(0.31, 0.75)** | **0.001** |
| Not reported | ~ | 56 | 1.03 |  |  |  |  |  |  |
| **cART duration** |  |  |  |  |  | <0.001 |  |  | **<0.001** |
| Prior to cART initiation | ~ | 35 | 3.12 | 1 |  |  | 1 |  |  |
| <6 months | ~ | 68 | 6.35 | 1.79 | (1.11, 2.88) | 0.018 | 1.16 | (0.70, 1.93) | 0.570 |
| 6-12 months | ~ | 24 | 1.70 | 0.57 | (0.32, 1.03) | 0.063 | **0.53** | **(0.29, 0.98)** | **0.043** |
| >12 months | ~ | 153 | 0.69 | 0.35 | (0.23, 0.52) | <0.001 | **0.42** | **(0.27, 0.64)** | **<0.001** |
| **Cotrimoxazole use** |  |  |  |  |  |  |  |  |  |
| No | ~ | 177 | 0.83 | 1 |  |  | 1 |  |  |
| Yes | ~ | 103 | 2.22 | 1.54 | (1.18, 2.01) | 0.001 | **0.65** | **(0.49, 0.87)** | **0.004** |
| **Isoniazid use** |  |  |  |  |  |  |  |  |  |
| No | ~ | 268 | 1.07 | 1 |  |  | 1 |  |  |
| Yes | ~ | 12 | 1.37 | 1.11 | (0.60, 2.06) | 0.740 | 0.99 | (0.53, 1.85) | 0.965 |
| ^a^ Global p-values for age, VL and CD4 are tests for trend. All other global p-values are tests for heterogeneity excluding missing values.  ^b^ Non-significant covariates were presented in the final model adjusted for the significant covariates, however they did not form part of the final model.  ^c^ P-values in bold represent significant covariates in the final model.  ^d^ ~ Viral load, CD4, BMI, cART duration, cotrimoxazole and isoniazid use are time-updated variables. | | | | | | | | | |

Supplementary Table 5: Factors associated with TB diagnosis in females

|  |  |  |  | **Univariate** | | | **Multivariate^b^** | | |
| --- | --- | --- | --- | --- | --- | --- | --- | --- | --- |
|  | **No of patients^d^** | **No of TB diagnosis** | **Rate (/100pys)** | **HR** | **95% CI** | **p-value^a^** | **HR** | **95% CI** | **p-value^a,c^** |
| **Total** | **2205** | **88** | **0.78** |  |  |  |  |  |  |
| **Age at TAHOD entry (years)** |  |  |  |  |  | 0.197 |  |  | 0.859 |
| ≤30 | 661 | 32 | 1.00 | 1 |  |  | 1 |  |  |
| 31-40 | 957 | 39 | 0.78 | 0.95 | (0.58, 1.54) | 0.826 | 1.17 | (0.71, 1.91) | 0.540 |
| 41-50 | 431 | 14 | 0.62 | 0.80 | (0.42, 1.54) | 0.502 | 1.14 | (0.58, 2.24) | 0.702 |
| >50 | 156 | 3 | 0.33 | 0.44 | (0.13, 1.47) | 0.181 | 0.61 | (0.18, 2.10) | 0.434 |
| **HIV Exposure** |  |  |  |  |  | 0.534 |  |  | 0.581 |
| Heterosexual contact | 2018 | 82 | 0.79 | 1 |  |  | 1 |  |  |
| Homosexual contact | 12 | 0 | 0.00 | N/A | N/A | N/A | N/A | N/A | N/A |
| Injecting drug use | 37 | 1 | 0.64 | 0.51 | (0.07, 3.89) | 0.518 | 0.55 | (0.07, 4.22) | 0.562 |
| Other/Unknown | 138 | 5 | 0.70 | 1.93 | (0.72, 5.18) | 0.192 | 1.90 | (0.69, 5.23) | 0.212 |
| **Hepatitis B co-infection** |  |  |  |  |  |  |  |  |  |
| Negative | 1556 | 58 | 0.70 | 1 |  |  | 1 |  |  |
| Positive | 128 | 4 | 0.64 | 1.11 | (0.40, 3.10) | 0.845 | 1.13 | (0.40, 3.19) | 0.813 |
| Not tested | 521 | 26 | 1.06 |  |  |  |  |  |  |
| **Hepatitis C co-infection** |  |  |  |  |  |  |  |  |  |
| Negative | 1436 | 54 | 0.70 | 1 |  |  | 1 |  |  |
| Positive | 112 | 1 | 0.17 | 0.26 | (0.04, 1.89) | 0.183 | 0.20 | (0.03, 1.51) | 0.119 |
| Not tested | 657 | 33 | 1.07 |  |  |  |  |  |  |
| **Prior TB** |  |  |  |  |  |  |  |  |  |
| No | 1888 | 67 | 0.69 | 1 |  |  | 1 |  |  |
| Yes | 317 | 21 | 1.33 | 1.21 | (0.73, 2.00) | 0.463 | 1.14 | (0.66, 1.98) | 0.638 |
| **Viral Load (copies/mL)** |  |  |  |  |  | <0.001 |  |  | **0.012** |
| <400 | ~ | 17 | 0.23 | 1 |  |  | 1 |  |  |
| 400-999 | ~ | 2 | 1.22 | 7.05 | (1.54, 32.21) | 0.012 | **5.76** | **(1.24, 26.61)** | **0.025** |
| 1000-4999 | ~ | 0 | 0.00 | N/A | N/A | N/A | N/A | N/A | N/A |
| ≥5000 | ~ | 20 | 2.07 | 6.80 | (3.17, 14.58) | <0.001 | **2.88** | **(1.25, 6.63)** | **0.013** |
| Not reported | ~ | 49 | 1.98 |  |  |  |  |  |  |
| **CD4 (cells/uL)** |  |  |  |  |  | <0.001 |  |  | **<0.001** |
| ≤50 | ~ | 14 | 7.13 | 1 |  |  | 1 |  |  |
| 51-100 | ~ | 5 | 2.24 | 0.44 | (0.16, 1.25) | 0.125 | 0.52 | (0.18, 1.50) | 0.227 |
| 101-200 | ~ | 19 | 2.03 | 0.43 | (0.21, 0.88) | 0.021 | 0.56 | (0.27, 1.17) | 0.122 |
| >200 | ~ | 44 | 0.45 | 0.11 | (0.06, 0.21) | <0.001 | **0.18** | **(0.09, 0.36)** | **<0.001** |
| Not reported | ~ | 6 | 3.54 |  |  |  |  |  |  |
| **BMI (kg/m^2^)** |  |  |  |  |  |  |  |  |  |
| <25 | ~ | 63 | 0.76 | 1 |  |  | 1 |  |  |
| ≥25 | ~ | 5 | 0.32 | 0.31 | (0.12, 0.77) | 0.012 | **0.40** | **(0.16, 1.01)** | **0.051** |
| Not reported | ~ | 20 | 1.30 |  |  |  |  |  |  |
| **cART duration** |  |  |  |  |  | <0.001 |  |  | **0.006** |
| Prior to cART initiation | ~ | 18 | 3.37 | 1 |  |  | 1 |  |  |
| <6 months | ~ | 16 | 3.36 | 1.63 | (0.74, 3.61) | 0.226 | 1.02 | (0.43, 2.41) | 0.970 |
| 6-12 months | ~ | 5 | 0.84 | 0.41 | (0.14, 1.18) | 0.099 | **0.34** | **(0.11, 1.06)** | **0.062** |
| >12 months | ~ | 49 | 0.50 | 0.29 | (0.16, 0.52) | <0.001 | **0.38** | **(0.20, 0.71)** | **0.003** |
| **Cotrimoxazole use** |  |  |  |  |  |  |  |  |  |
| No | ~ | 54 | 0.57 | 1 |  |  | 1 |  |  |
| Yes | ~ | 34 | 1.89 | 2.23 | (1.41, 3.51) | 0.001 | 0.96 | (0.55, 1.67) | 0.886 |
| **Isoniazid use** |  |  |  |  |  |  |  |  |  |
| No | ~ | 85 | 0.77 | 1 |  |  | 1 |  |  |
| Yes | ~ | 3 | 1.10 | 1.76 | (0.50, 6.22) | 0.382 | 1.80 | (0.49, 6.58) | 0.372 |
| ^a^ Global p-values for age, VL and CD4 are tests for trend. All other global p-values are tests for heterogeneity excluding missing values.  ^b^ Non-significant covariates were presented in the final model adjusted for the significant covariates, however they did not form part of the final model.  ^c^ P-values in bold represent significant covariates in the final model.  ^d^ ~ Viral load, CD4, BMI, cART duration, cotrimoxazole and isoniazid use are time-updated variables. | | | | | | | | | |
